# Pathophysiology, Clinical Heterogeneity, and Therapeutic Advances in Amyotrophic Lateral Sclerosis: A Comprehensive Review of Molecular Mechanisms, Diagnostic Challenges, and Multidisciplinary Management Strategies

**DOI:** 10.3390/life15040647

**Published:** 2025-04-14

**Authors:** María González-Sánchez, María Jesús Ramírez-Expósito, José Manuel Martínez-Martos

**Affiliations:** Experimental and Clinical Physiopathology Research Group CTS-1039, Department of Health Sciences, School of Health Sciences, University of Jaén, E23071 Jaén, Spain; mgs00080@red.ujaen.es (M.G.-S.); mramirez@ujaen.es (M.J.R.-E.)

**Keywords:** amyotrophic lateral sclerosis, neurodegenerative disease, *C9orf72* expansion, TDP-43 proteinopathy, *SOD1* mutations, multidisciplinary care, riluzole, non-invasive ventilation, frontotemporal dementia, neurofilament light chain

## Abstract

Amyotrophic lateral sclerosis (ALS) is a progressive neurodegenerative disorder characterized by the progressive degeneration of upper and lower motor neurons, leading to muscle atrophy, paralysis, and respiratory failure. This comprehensive review synthesizes the current knowledge on ALS pathophysiology, clinical heterogeneity, diagnostic frameworks, and evolving therapeutic strategies. Mechanistically, ALS arises from complex interactions between genetic mutations (e.g., in *C9orf72*, *SOD1*, *TARDBP* (TDP-43), and *FUS*) and dysregulated cellular pathways, including impaired RNA metabolism, protein misfolding, nucleocytoplasmic transport defects, and prion-like propagation of toxic aggregates. Phenotypic heterogeneity, manifesting as bulbar-, spinal-, or respiratory-onset variants, complicates its early diagnosis, which thus necessitates the rigorous application of the revised El Escorial criteria and emerging biomarkers such as neurofilament light chain. Clinically, ALS intersects with frontotemporal dementia (FTD) in up to 50% of the cases, driven by shared TDP-43 pathology and *C9orf72* hexanucleotide expansions. Epidemiological studies have revealed a lifetime risk of 1:350, with male predominance (1.5:1) and peak onset between 50 and 70 years. Disease progression varies widely, with a median survival of 2–4 years post-diagnosis, underscoring the urgency for early intervention. Approved therapies, including riluzole (glutamate modulation), edaravone (antioxidant), and tofersen (antisense oligonucleotide), offer modest survival benefits, while dextromethorphan/quinidine alleviates the pseudobulbar affect. Non-pharmacological treatment advances, such as non-invasive ventilation (NIV), prolong survival by 13 months and improve quality of life, particularly in bulb-involved patients. Multidisciplinary care—integrating physical therapy, respiratory support, nutritional management, and cognitive assessments—is critical to addressing motor and non-motor symptoms (e.g., dysphagia, spasticity, sleep disturbances). Emerging therapies show promise in preclinical models. However, challenges persist in translating genetic insights into universally effective treatments. Ethical considerations, including euthanasia and end-of-life decision-making, further highlight the need for patient-centered communication and palliative strategies.

## 1. Introduction

The term “amyotrophic lateral sclerosis” (ALS) was designated by Jean-Martin Charcot, a French neurologist, in the 19th century [1]. The concept of “lateral sclerosis” symbolizes the scarring of the lateral spinal cord tissues, and the word “amyotrophic” stands for muscle atrophy.

ALS is a heterogeneous, adult-onset, fatal neurodegenerative disorder that primarily affects the motor system [2]. This disease causes the upper motor neurons to degenerate in the motor cortex and the lower motor neurons to degenerate in the brainstem and spinal cord, resulting in progressive motor impairment with muscle weakness, spasticity, and atrophy [3].

Extramotor involvement with behavioral and cognitive impairment due to anterior temporal and frontal neuronal loss is present in up to 50% of the patients [3]. This disease is considered a highly genetically heterogeneous syndrome. In fact, the predominant mutant protein in up to 97% of patients is TAR DNA-binding protein 43 (TDP-43) [2].

From a neuroanatomical perspective, ALS has been strongly linked to the corticospinal system [4], with early dysfunction and loss of Betz cells [5]. There are extensive corticofugal projection neurons that span the corticomotoneuronal system exhibiting monosynaptic connections with bulbar and spinal motor units [4,6,7]. These projections are central to the execution of a series of tasks that involve bulbar and upper and lower limb activities, tasks that are compromised in the early stages of the pathology. Therefore, we can consider this disease a uniquely human form of neurodegeneration, due in its beginnings to a systemic failure, specifically, a failure of the human corticomotoneuronal system. This failure progresses over the years and is strongly linked to the effects mediated by genes, aging, the epigenome, and the exposome of ALS.

## 2. Clinical Manifestations

The clinical manifestations of upper motor neuron damage manifest primarily with increased muscle tone (spasticity), clumsy movements, positive pathological signs, active or hyperactive tendon reflexes, and pseudobulbar palsy, while the clinical manifestations of lower motor neuron damage manifest mainly with muscle atrophy, muscle weakness, decreased muscle tone, muscle twitching, and decreased tendon reflexes [8,9,10,11,12,13].

This pathology begins in the extremities. Its detection may be lateralized, and the deficits are typically unilateral. These observations result in the pathologic process underlying the clinical symptoms being focal and randomized in the nervous system [14,15,16]. After this event, the disease thrives by contiguity, showing itself in two ways: initially, motor dysfunction in a focal body region where symptoms are initiated worsens dramatically over time in that same area, and second, motor dysfunction spreads outward to contiguous regions and progresses from one side of the body to the other. This suggests that the pathology spreads neuroanatomically, starting within one area of the neuroaxis and then spreading to contiguous regions [14,15,16]. Likewise, symptoms that begin in the head, trunk, arm, or legs are also characteristic of upper and lower motor neuron dysfunction and are characterized as maximal in these same regions. This means that the area where the first symptoms appear is the area that has maximal upper and lower motor neuron dysfunction degeneration, suggesting that at the onset of ALS, these upper and lower motor neuron dysfunction degenerations are connected, i.e., they are dependent on each other. As the pathology progresses contiguously, degeneration will proceed independently at the level of the upper and lower motor neurons.

Therefore, biomarkers that reliably reflect pathology-specific particularities that reinforce an early diagnosis and promote the identification of outcome parameters for clinical trials are rapidly required to obtain progress in all therapies [17,18].

Specifically, the noninvasive biomarker method called neuroimaging potential has been endorsed by the ALS research community globally [17,19,20,21]. The potential of neuroimaging relies on detecting the hidden involvement of upper and lower motor neurons through the examination of neuronal white matter. In fact, diffusion tensor imaging metrics have shown significant white matter alterations in the corticospinal tracts in ALS [22].

## 3. Genetics

ALS is currently classified as familial ALS and sporadic ALS. Familial ALS, which constitutes 10–15% of the cases, is defined as inheritance among family members of ALS and associated syndromes [8,9].

About 70% of the familial cases have mutations in known ALS genes acquired by autosomal dominant, autosomal recessive, and X-chromosomal inheritance. Sporadic ALS accounts for 85% of the cases and is defined as a disease occurring in patients with no family history of ALS. About 15% of the patients with sporadic ALS carry pathogenic mutations in known ALS genes, i.e., mutations limited to a single subject; so they have no family history of ALS [8,9]. The etiology of ALS in the 85% of sporadic ALS cases is unknown.

Initially, this pathology was considered a disease characterized by motor dysfunction. However, it is now recognized that different behavioral and cognitive changes occurring early in the course of the disease [23,24] occur in a percentage of ALS patients ranging from 35 to 50% [25,26].

Although the pathophysiological mechanisms of ALS are not fully understood, the influence of genetic factors on ALS development has been highly recognized in recent years. The genetic architecture of this disease is very complex. However, at present, more than forty causal genes associated with ALS have been identified [27,28]. The most common pathogenic genes among them are *C9orf72*, *TARDBP* (TDP-43), *SOD1*, and *FUS*.

In recent times, progressive evidence has indicated that signs of frontotemporal dementia are initially seen in patients diagnosed with ALS, implying a clinical overlap between these two disorders [29,30,31]. This finding has resulted in determining that ALS and frontotemporal dementia share a common biology and exist in the same pathological spectrum. Up to 50% of ALS patients have a degree of cognitive or behavioral impairment, and up to 33% of frontotemporal dementia patients have evidence of motor neuron involvement [32,33,34,35,36,37]. In addition, there is evidence of neuropathological features occurring at the molecular level: >95% of the nervous system in ALS patients and 50% in frontotemporal dementia patients share the common molecular signature of TDP-43 proteinopathy [38,39,40,41]. Also, the most common genetic cause of ALS and frontotemporal dementia is *C9orf72* mutation, which can result in either pathology or in a combination of both [42,43].

People with ALS show loss of normal language and executive function. Generally, long-term memory remains intact [26]. In addition, other repercussions of the disease are irritability, apathy, disregard for hygiene, and changes in the eating habits. Fifteen percent of the affected subjects meet the diagnostic criteria for frontotemporal dementia [24]. Pseudobulbar involvement, which is considered a brain problem characterized by sudden uncontrollable bouts of crying or laughing in situations where emotion is magnified or where these two reactions are not expected, combined with anxiety, depression, and sleep disruptions, causes emotional lability [44]. Because of these behavioral as well as cognitive changes, ALS is a global neurodegenerative disease.

Frontotemporal dementia disease is associated with neurodegeneration that manifests as macroscopic atrophy of the frontal and temporal lobes [45,46]. In such patients, the frontal and temporal areas show a reduction and a spongiform morphology due to extensive neuronal loss. ALS cases with cognitive impairment due to frontotemporal dementia show signs of spongiform degeneration in the frontal and precuneus gyrus and diffuse subcortical glycolysis [36,37,47]. In addition, neuronal loss can be seen in the anterior cingulate gyrus, as well as in the substantia nigra and amygdala [36,37]. In general, neuronal degeneration is progressive and increases during the course of the pathology, although it may actually vary from one individual to another. Clinically, frontotemporal dementia has three predominant clinical phenotypes: the primary progressive aphasia, the semantic dementia, and the behavioral variants.

Like the ALS clinical phenotypes, the frontotemporal dementia phenotypes are largely determined by the anatomic distribution of the pathology in the early disease [48]. Thus, the frontotemporal dementia phenotypes can be viewed as a focally initiated pathology.

## 4. Epidemiology

The lifetime risk of amyotrophic lateral sclerosis is minimal, approximately 1/350 [49,50]. Its prevalence varies from 5 to 8 per 100,000 individuals, and the onset of the pathology peaks between 50 and 70 years of age [51,52].

However, the lifetime risk of ALS is 1:400 for women and 1:300 for men [53,54]. Furthermore, the median survival is reported to be 2.5 to 3.5 years after symptom onset and 1.5 to 2.5 years after disease diagnosis [55,56,57].

The incidence of ALS is highest at the age of 60–79 years [58,59] and increases progressively with age. Some studies show stability of the ALS incidence in recent decades [49], while others observe a certain increase [60,61]. The reason for these differences could be the use of better diagnostics [62].

The best epidemiological data at the national level come mainly from Western Europe or countries with a majority of European migrant populations. In European studies, the incidence is 1.47 per 100,000 individuals, and in most studies, it ranges from 1.0 to 3.0 per 100,000 people [63]. In Serbia, the annual incidence is 1.11/100,000 people/year, in Denmark 5.55/100,000/year, in Sardinia 1.33/100,000/year, and in Liguria (Italy) 3.22/100,000/year. The lowest incidence rates are in Serbia [64], Russia [65], and Cyprus [66,67].

The global standardized incidence of ALS established by a meta-analysis is 1.68 per 100,000 persons, depending on the location. The prevalence in Western Europe was estimated at 4.06 per 100,000 persons [63]. In North America, incidences around 1.75 per 100,000 persons have been estimated [63]. In Asian populations, it ranges from 0.73 per 100,000 people in South Asia to 0.94 per 100,000 people in West Asia, while Oceania has one of the highest incidence rates [68].

In South America, the annual incidence is 0.26/100,000 persons/year in Ecuador, 1.40/100,000/year in Colombia, and 3.17/100,000/year in Argentina [69].

The U.S. National ALS Registry reports a U.S. prevalence estimate of 3.9 per 100,000 individuals from 2010 to 2011 [70,71], and in general, people who are male, white, non-Hispanic, and older than 60 years possess a higher likelihood of developing ALS [59]. Thus, the differences between Western Europe and the United States are small [72].

Meanwhile, the incidence and prevalence of ALS in different continents show greater variation. In Africa, the average annual crude incidence is 1.01–1.22/100,000 persons/year. In Asia, it varies from 0.42/100,000/year in Iran to 2.20/100,000/year in Japan. The highest incidence rate occurred in the Kii peninsula. Notably, the prevalence of upper motor neuron diseases is higher than that of lower motor neuron diseases in Gujarat, India [60,73].

In addition, the impact of this pathology varies according to sex, with an overall male/female ratio of 1.35. Another significant factor is genetics; heritability is higher in mother–daughter pairs, while mutations in *C9orf72*, the ALS risk gene, cause a reduction in the age of onset in men compared to women. Mutation of this gene accounts for 30–40% of the ALS cases in the United States and Europe. *C9orf72* (i.e., open reading frame gene 72 on chromosome 9) contains a hexanucleotide repeat expansion (GGGGCC) responsible for ALS and frontotemporal dementia. This gene encodes a protein found in many tissues that forms a complex with SMCR8 and WDR41, which regulates vesicle trafficking, lysosome homeostasis, mTORC1 signaling, and autophagy [50,74,75]. Therefore, the existence of regional variations could be considered.

## 5. Pathophysiology

The pathophysiology of ALS is still not fully understood. As the genetic architecture of this disease is researched and understood, the mechanisms by which the functions of certain ALS mutations converge in highly recurrent nervous system pathological pathways are being discovered [76,77].

Common pathological pathways in this disease include altered autophagy, insufficient RNA metabolism, mitochondrial dysfunction, cytoskeleton defects, and DNA repair impairment [78,79].

Common ALS gene alterations include *C9orf72*, *TARDBP*, and *FUS* mutations, which alter RNA metabolism, and *C9orf72* repeat expansions, which induce proteostasis defects, mitochondrial dysfunction, and oxidative stress [78].

The genetic diagnostic method demonstrates the lack of heritability in ALS patients. Some increase in the levels of rare variants was established, e.g., in those of untranslated regions of disease-causing genes, including *SOD1*, *TARDBP*, *FUS*, *VCP*, *OPTN*, and *UBQLN2*, highlighting the importance of regions that determine the pathogenesis of this pathology [80].

### 5.1. Defects in Nucleocytoplasmic Transport in ALS

**Figure 1:**
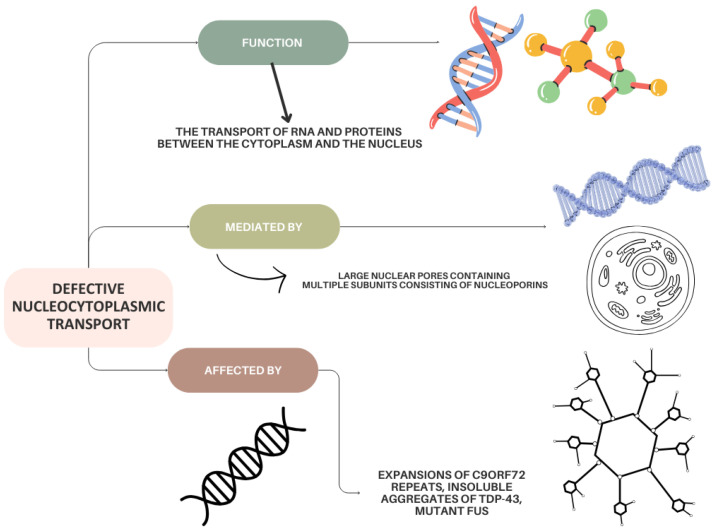
Spatial organization of nucleoporins in the nuclear pore complex.

Nucleocytoplasmic transport is a highly regularized process whose function is the transport of RNA and proteins between cytoplasm and nucleus [81]. It is carried out through large nuclear pores containing multiple protein subunits consisting of nucleoporins, whose function is to act in concert with cytoplasmic importins (which import protein cargoes from the cytoplasm to the nucleoplasm) and nuclear exportins (which export protein cargoes from the cytoplasm to the nucleoplasm) [77,81,82,83] (Figure 1). The direction of this transport is determined by small Ras-linked nuclear proteins via binding of importins and exportins. Nucleocytoplasmic transport morphology and the nuclear envelope are affected by expansions of *C9orf72* repeats [84,85], insoluble aggregates of TDP-43, and mutant *FUS* [86]. Abnormal immunoreactivity against nucleoporins, importins, and Ran in motor cortex and spinal motor neurons in sporadic ALS and mutant *TARDBP*, in addition to *C9orf72* repeat expansions [87,88], can be readily detected in individuals with this pathology.

### 5.2. C9orf72 Dipeptide Repeat Proteins and Neurotoxicity

**Figure 2:**
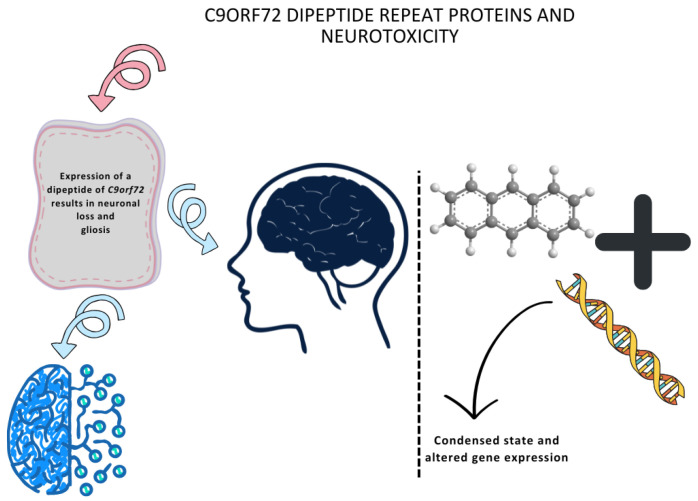
Poly(PR) and poly(GR) enhance FUS aggregation and phase separation.

The expression of poly(PR), a dipeptide of *C9orf72*, results in neuronal loss and gliosis, leading to motor and memory deficits [89,90,91]. Poly(PR) binds to the DNA and colocalizes with heterochromatin, leading to an altered condensed state and altered gene expression (Figure 2).

### 5.3. Liquid–Liquid Phase Separation

**Figure 3:**
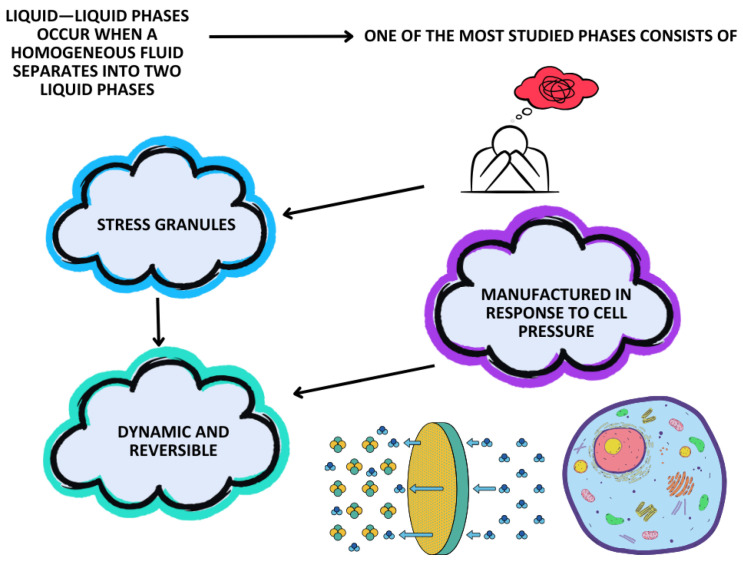
ALS-related mechanisms, associated or not with PSLL. Schematic illustration of the most important ALS-related pathological process that depends on or is influenced by the functioning of PSLL in motor neurons.

Liquid–liquid phases occur when a homogeneous fluid separates into two liquid phases, forming a dynamic structure equal to an organelle that lacks a membrane [92]. These phases are linked to a variety of pathophysiological processes in ALS, including RNA metabolism, DNA repair, and axonal transport [92]. One of the most studied phases consists of stress granules, which are manufactured in response to cell pressure; however, stress granules are dynamic and reversible once a cell is shrinking. In ALS, stress granules are affected, which results in more persistent granules and the formation of RNA and protein aggregates, such as TDP-43 and *FUS* granules [92,93] (Figure 3).

### 5.4. Cell-to-Cell Prion Transmission

**Figure 4:**
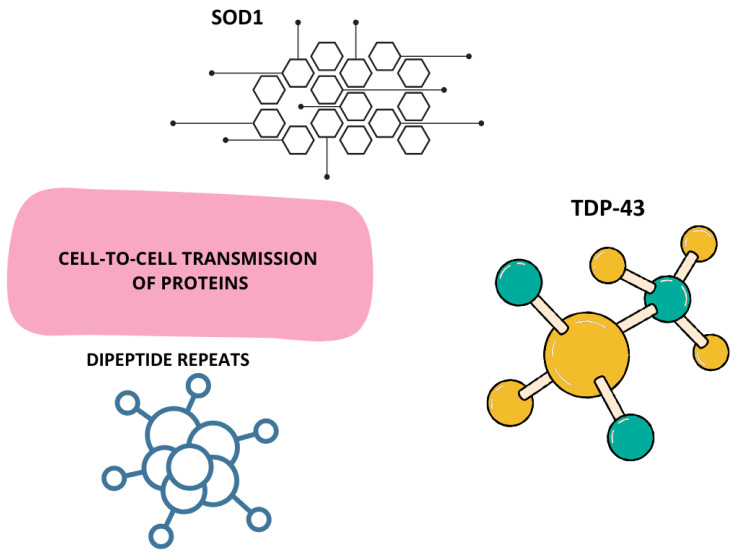
Graphic summary of prion transmission between cells.

The low-complexity domains of TDP-43 and *FUS* contain prion-like motifs [92]. A research focus of studies on the development of ALS is the cell-to-cell transmission of proteins that are predisposed to aggregation, including *SOD1* [94], dipeptide repeats [95,96], and TDP-43 [16,97,98] (Figure 4).

### 5.5. ALS Biomarkers

**Figure 5:**
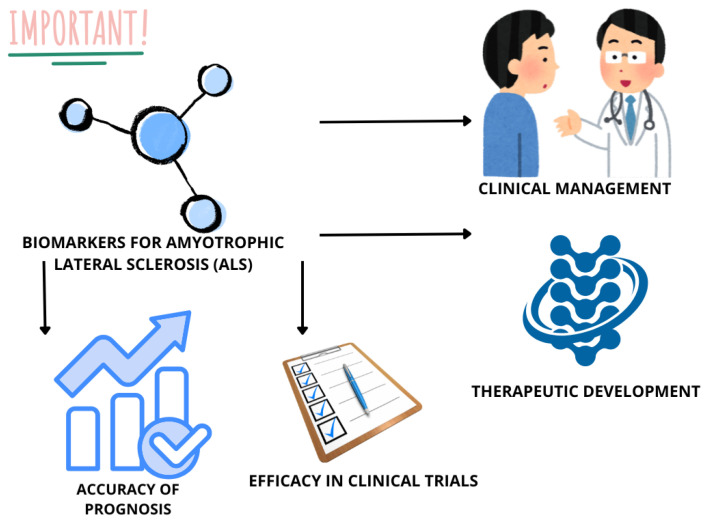
Importance of ALS biomarkers.

The identification of biomarkers for ALS (Figure 5) allows for advancing both clinical management and therapeutic development. Reliable biomarkers could enable the early diagnosis of ALS, which is currently limited by the disease’s heterogeneity and lack of definitive diagnostic tools. Additionally, biomarkers may serve as objective measures of disease progression, enhancing the accuracy of prognosis and the evaluation of therapeutic efficacy in clinical trials [76,99,100].

#### 5.5.1. Neurofilaments

**Figure 6:**
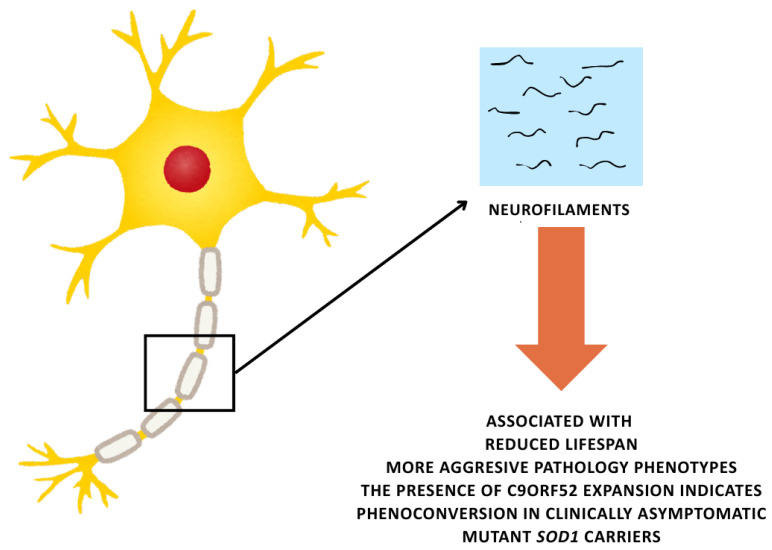
Temporal trajectory of the effects of neurofilament NfL expression and its utility as a biomarker through the course of pre-symptomatic and symptomatic ALS.

Neurofilament light chain (NfL) remains the most validated biomarker for ALS, with its elevated CSF and serum levels correlating with disease progression rate (ρ = 0.72) and survival [101,102] (Figure 6). Plasma neurofilaments are also associated with reduced lifespan, more aggressive pathological phenotypes, and the presence of C9orf52 expansion [103,104]; their levels are elevated up to five years before disease onset in sporadic and familial ALS [105,106], and their presence indicates phenoconversion in clinically asymptomatic mutant *SOD1* carriers [105]. Recent studies highlight phosphorylated neurofilament heavy chain (pNfH) as a superior prognosticator in bulbar-onset ALS, showing 89% specificity in 12-month mortality prediction [107]. Glial fibrillary acidic protein (GFAP), a marker of astroglial activation, predicts cognitive decline in *C9orf72* carriers [108]. Liquid biopsy assays detecting TDP-43 fragments in exosomes now achieve 94% diagnostic accuracy in pre-symptomatic *C9orf72* carriers [109]. Emerging techniques, such as single-molecule array (SIMOA) for ultrasensitive NfL quantification, enable the stratification of therapeutic responders in clinical trials [110].

#### 5.5.2. Brain Imaging

**Figure 7:**
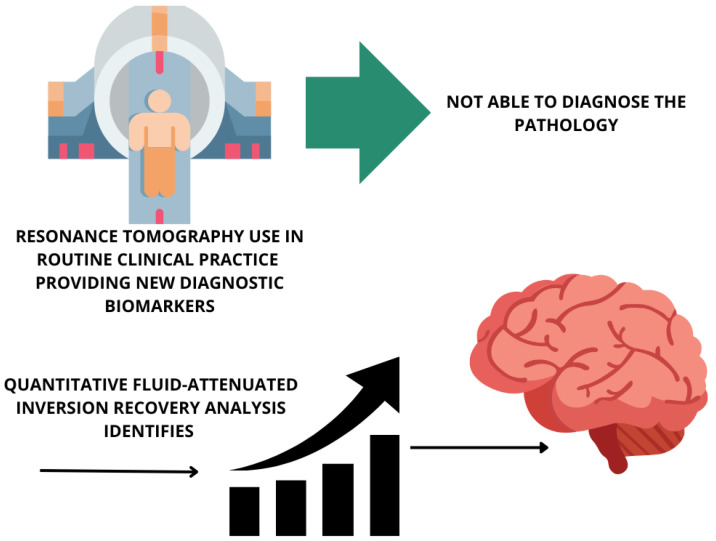
Neuroimaging biomarkers in cognitively normal older adults. Cognitively normal older adults can present with varying amounts of amyloid and tau positivity.

Magnetic resonance tomography does not allow for the diagnosis of this pathology, but quantitative fluid-attenuated inversion recovery analysis identifies increased signal intensities in the corticospinal tract and corpus callosum of ALS patients [111]. Newer structural and functional magnetic resonance tomography techniques are in routine clinical practice, providing new diagnostic biomarkers (Figure 7).

#### 5.5.3. Emerging Biomarkers

Beyond neuroimaging and neurofilament light chain (NfL), several promising biomarkers are being investigated to improve the diagnosis and prognosis of ALS as well as the monitoring of treatment response. These emerging biomarkers span multiple biological compartments and reflect diverse aspects of ALS pathology. While NfL is a well-established biomarker for neurodegeneration, its lack of disease specificity has spurred the search for more targeted markers. Several studies have focused on proteins in the plasma [109,112] and cerebrospinal fluid (CSF) [113,114] related to specific ALS-associated genes and pathological processes. Additionally, alterations in the levels of proteins involved in RNA metabolism, such as hnRNP A1, are being explored as potential biomarkers [115,116].

MicroRNAs (miRNAs), small non-coding RNA molecules, play a critical role in gene regulation and are implicated in ALS pathogenesis. Dysregulation of specific miRNAs has been observed in the plasma, CSF, and affected tissues of ALS patients. Certain miRNAs, such as miR-206, miR-214, miR 374n-5p, and miR-143-3p, have shown potential as diagnostic and prognostic biomarkers in ALS [117,118].

Exosomes are extracellular vesicles that mediate intercellular communication and contain a cargo of proteins, nucleic acids, and lipids. Exosomes derived from motor neurons and glial cells can be isolated from the plasma and CSF and analyzed for ALS-related biomarkers. Studies have demonstrated that exosomes from ALS patients contain misfolded proteins, such as *SOD1* and TDP-43, and altered miRNA profiles [119]. Exosomal biomarkers hold promise for capturing disease-specific molecular signatures and monitoring disease progression.

Given the primary involvement of the muscles in ALS pathology, muscle-derived biomarkers are being investigated as potential indicators of disease activity and treatment response. Muscle biopsies can be analyzed for gene expression profiles, protein levels, and pathological changes. Non-invasive techniques, such as quantitative muscle ultrasound and magnetic resonance spectroscopy, are also being explored to assess muscle structure and function [120,121].

With the rise of wearable sensors and mobile health technologies, digital biomarkers are emerging as a novel approach to monitor disease progression and treatment response in ALS. These biomarkers include measures of motor function, speech, sleep, and cognitive performance collected remotely and continuously. Digital biomarkers offer the potential for a more frequent and objective assessment of disease activity in ALS patients [122,123,124,125].

## 6. Etiopathogenesis

The causes of this pathology are considered unknown for most patients. ALS is based on the degeneration of upper and lower motor neurons, but there is some uncertainty as to whether the same pathogenesis is identical in all cases. Thus, this pathology could be considered a multifactorial pathology in which the interaction between genetic background and external factors plays an important role.

The main risk factor for ALS is male sex. Male human beings possess a 1.5 times higher risk for developing this disease compared to females [59,70]. However, after menopause, the incidence of ALS becomes almost equal between the genders due to female hormones such as estrogen and progesterone being protective factors against ALS triggers. A case–control study in the Netherlands between 2006 and 2009 showed that endogenous exposure to estrogen throughout life is associated with increased survival in patients with this disease [126].

The increased risk of this pathology in males could be due to the sex hormone testosterone. Indeed, testosterone begins to affect the individual in utero, and it has been suggested that fetal testosterone is a risk factor for ALS, with anthropomorphic measures supporting this connection. Notably, the relative length of the second and fourth fingers is significantly related to the fetal testosterone level [127], and indeed, ALS patients are characterized by a relative difference in finger length that is statistically greater than the general population’s mean difference [128]. In addition, over time, they are more likely to be exposed to many environmental risk factors linked to their profession such as physical activity, head traumas, heavy metals, or other occupational hazards.

Smoking has been shown in several studies to increase the risk of ALS [129]. It is not known for sure whether the association between ALS and smoking is due to nicotine, oxidative stress, or toxic substances in tobacco smoke [130].

Occupational hazards related to many types of jobs are proposed as being predisposing to ALS and are associated with jobs that require physical activity and have a high risk of trauma [131]. No causal factors have been identified. Several studies confirmed that military service in the United States is a risk factor for ALS [132]. Specifically, the risk is 0.22 [133], with a standardized mortality of 1.92 [134]. Military service is also linked to exposure to strenuous physical exertion, sleep deprivation, trauma, psychological stress, and lead. In addition, there does not appear to be a general association between ALS and military service [135].

Physical exercise has been positively associated with ALS incidence in many studies, but not all [136,137]. A dose relationship between physical exercise and ALS has not resulted, and it has been observed that the greatest risk is not physical activity per se, but unknown congenital factors predisposing to ALS individuals that perform physical activity [138]. Head trauma can cause a neurodegenerative disease called chronic traumatic encephalopathy [139,140].

## 7. Diagnosis

At the onset of this pathology, it is very unlikely that ALS patients visit a neurologist for diagnostic suspicion [141,142]; so, a low threshold for neurological referral is needed when patients present dysphagia (difficulty swallowing), limb weakness, muscular respiratory insufficiency, and progressive dysarthria (are weak or have difficulties in controlling the speech muscles). ThinkALS is a tool of the ALS Association that promotes the early neurological referral to avoid unnecessary procedures and allow patients to enter pathology-modifying treatments to increase patient enrollment in clinical trials [143].

**Figure 8:**
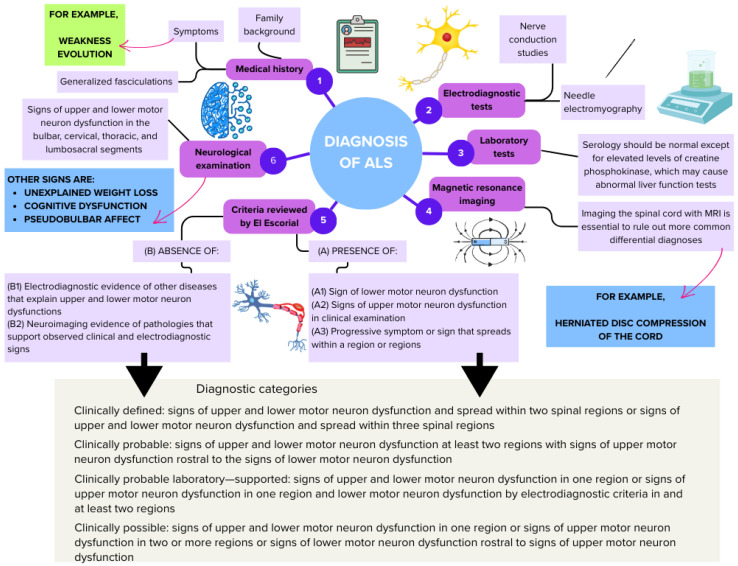
Diagnostic algorithm for amyotrophic lateral sclerosis (ALS) diagnosis. The flowchart illustrates the stepwise diagnostic process for ALS, starting with the assessment of clinical history (1) that includes family history and the identification of the initial symptoms. Electrophysiological tests (2), such as nerve conduction studies and needle electromyography, are indicated, along with laboratory tests (3) to rule out other conditions. Magnetic resonance imaging (MRI) (4) of the spinal cord is crucial to exclude differential diagnoses like a herniated disc or spinal cord compression. The revised El Escorial criteria (5) are fundamental to confirm the presence (A) or absence (B) of specific signs of upper and lower motor neuron dysfunction, evaluated during the neurological examination (6). The figure summarizes the diagnostic categories ranging from “clinically definite” to “clinically possible,” based on the combination of clinical signs, electrodiagnostic findings, and neuroimaging results. Additional symptoms include unexplained weight loss, cognitive dysfunction, and pseudobulbar affect.

Clues that, in sum, point to a diagnosis of ALS include unexplained weight loss, changes in cognition, generalized fasciculations, executive dysfunctions (dysfunctions in routines responsible for monitoring cognitive processes during the performance of complex cognitive tasks), family history of ALS, and pseudobulbar affect. Clinical features that do not support this pathology include autonomic nervous system dysfunction, anterior visual abnormalities, sensory and sphincter dysfunction. Clinical history recording and neurological examination are accompanied by serological and electrodiagnostic tests [13]. These patients usually have normal serology, except for high creatine phosphokinase levels in several cases. Nerve conduction studies override motor nerve conduction obstruction and sensory nerve involvement. Needle electromyography allows for verifying lower motor neuron involvement [144,145] (Figure 8).

A large number of neurologists use the revised El Escorial criteria (summarized in Figure 1) to subclassify ALS as possible, probable, probable–laboratory-supported, and defined ALS, depending on clinical presentation and electromyographic findings [146]. In general, an early diagnosis of ALS is important. As simplified diagnostic criteria become more widely accepted, practitioners will recognize and treat ALS in the early stages of the disease (Figure 1).

The onset of this disease is misleading and can repeat a wide variety of certain conditions, which can sometimes lead to delays in its diagnosis and misclassification [147]. Establishing a good doctor–patient relationship before diagnosis confirmation occurs is crucial because it reduces the patient’s fears and dispels erroneous thoughts such as “I can’t do anything” or that the conclusion of this disease is to die of asphyxiation [148].

On the other hand, it is considered very necessary to assess cognition in patients with this pathology, even though cognitive impairment does not fall within the formal ALS diagnostic criteria, because the cognition status is related to prognosis and rate of progression and, so, informs the clinical management [25,26]. The assessment of cognitive impairment in ALS patients should include social cognition, executive dysfunction, and language dysfunction [149]. Dehydration, loss of empathy, and apathy, which are considered behavioral impairments, affect the well-being of patients and family members and require evaluation.

The ALS Cognitive Behavioral Questionnaire, which is available in three languages, identifies cognitive and behavioral impairments [149].

The ALS-FTD Questionnaire (ALS-FTD-Q) is completed by healthcare professionals or caregivers to assess the behavioral changes in ALS patients [149]. The Beaumont Behavioral Inventory is a novel screening tool to assess behavioral impairment in ALS patients and may be more sensitive than the ALS-FTD-Q [149].

In general, recognizing cognitive symptoms is critical, as they are considered a manifestation of this pathology, and the proper identification of these symptoms improves ALS treatment, prognosis, and counseling. Since cognitive symptoms may change as the disease progresses, it is imperative to assess them periodically to improve patient care [150].

## 8. From Symptom Onset to Disease Progression

### 8.1. ALS in the Early Stage

In this first phase, individuals present with fatigue, mild but advanced muscle weakness, and performance restriction [151]. The onset of this disease occurs with asymmetrical limb weakness in specific muscles of both the upper and the lower body. The characteristic presentation of classic bulbar-onset ALS affects both upper and lower motor neurons, and the symptoms typically begin with dysarthria and then dysphagia and eventually spread to the extremities [8,152].

Lower body frailty is sometimes associated with unilateral or bilateral foot drop and, in effect, increased ambulation effort as the patient compensates with circumductal gait to avoid stumbling or falling. In addition, there may be weakness in the proximal part of the legs, which hinders load transfers both when sitting and when standing up. On the other hand, it should be noted that muscle weakness may be aggravated by spasticity. Together, these factors increase energy expenditure and lead to an increased risk of falls [153,154,155].

The rehabilitation evaluation in these cases begins with an initial assessment of gait, balance, manual motor strength, range of motion, and tone in order to study the risk of falls. It should be noted that a fall would cause great functional impairment; so, prevention is a priority in the treatment of this disease. For this purpose, orthoses can be used intermittently at the onset of the disease, when weakness is still mild [153,154].

Upper body weakness ranges from proximal weakness of the shoulder muscles to distal weakness that includes the wrist and hand muscles. This event interferes with the performance of activities of daily living. A variety of adaptive equipment is used in this case [151]. On the other hand, stretching exercises and those that focus on extending the joint range are paramount in the presence of the restless and spasmodic leg syndrome [156].

### 8.2. ALS in the Middle Phase

At this stage, weakness has increased exponentially in the lower extremities, resulting in great difficulty in making transfers (e.g., from sitting to standing). In such cases, transfer equipment such as elevating cushions or sliding boards are available. ALS will eventually require the use of assistive devices for ambulation and eventually the transition to a wheelchair. This stage also presents several types of symptoms such as musculoskeletal symptoms, spasticity, cramps, dysphagia, sialorrhea, fatigue and sleepiness, dysarthria, and weakness of the respiratory muscles [157].

#### 8.2.1. Musculoskeletal Symptoms

Over time, the patients may develop pain as a secondary complication of musculoskeletal dysfunction due to spasticity, poor mobility, loss of range of motion. Many ALS patients report pain even in the early stages of the disease [158,159], and pain is linked to reduced quality of life [158].

Surprisingly, little pain management strategies have been reported for ALS to date, though many causes of pain could be prevented with multiple interventions. The most common areas of pain are in the neck, shoulders, and low back. In addition, shoulder subluxations and contractures are very common in ALS patients [160]. In these cases, a regular stretching program could prevent shoulder pain.

In the lower extremities, discomfort may be combined with spasticity, edema, contractures, and loss of movement. In this case, edema can be reduced or disappear by manual therapy, leg elevation, and the use of compression stockings [144,161].

#### 8.2.2. Spasticity and Cramps

Treatment for spasticity in ALS is not very conclusive, although agents such as baclofen, tizanidine, benzodiazepine, and cannabinoids are available for use. In addition to these drugs, dalfampridine, a sustained-release form of 4-aminopyridine (4-AP) that is intended to restore conduction in focally demyelinated axons and inhibit voltage-gated potassium (KV) channels, can be used. 4-AP crosses the blood–brain barrier easily and has shown particular efficacy against fatigue, cognition impairment, and gait speed reduction [162]. These pharmacological properties have led to extensive research into its therapeutic potential for symptom management in patients with neuromuscular transmission disorders and demyelinating diseases such as spasticity in hereditary spastic paraparesis.

In vitro, various neuroprotective effects of 4-AP have been observed, such as increased levels of neuronal activity, caspase activation, reduction in endoplasmic reticulum stress, and correction of ion channel imbalances in motor neurons and induced pluripotent stem cells from patients with amyotrophic lateral sclerosis carrying mutations in the FUS and SOD1 genes [162]. Non-pharmacological techniques can also be used, but they are limited and temporary [163]. Cramps can be quite annoying in this pathology. Stretching, gentle exercise, and good hydration can be beneficial in these cases [164].

#### 8.2.3. Dysphagia and Sialorrhea/Secretion Management

Dysphagia and its development are predictable in this disease. Oropharyngeal and tongue weakness hinders both chewing and swallowing, and if coupled with sialorrhea, decreased ability to swallow saliva predominates. Symptoms of dysphagia include throat clearing, choking episodes, and coughing during and after meals. Undoubtedly, a negative prognostic factor in ALS is dysphagia, for the reason that it can lead to aspiration and malnutrition [147,165].

Saliva drooling is caused by oropharyngeal weakness, which makes it difficult to expectorate sputum and bronchial secretions. The fact that saliva escapes from the corner of the lips and, at the same time, the sensation of having dry secretions in the back of the throat are very unpleasant and frustrating for these individuals. The treatment, in this case, can be pharmacological but is counterproductive due to the great side effects that it has. Mucolytics can be used to counteract the problem [166].

#### 8.2.4. Fatigue and Sleepiness

The term fatigue refers to both the inability to maintain motor function during exertion and generalized tiredness [167]. The causes of fatigue are various, ranging from nocturnal hypoventilation to pain and cramps that disrupt sleep [168,169].

#### 8.2.5. Dysarthria

The fact that these patients lose the ability to communicate is very frustrating. Dysarthria can manifest at the onset of the disease or as a symptom associated with disease progression affecting the bulbar muscles [170,171]. It can be spastic, flaccid, or mixed, depending on upper or lower motor neuron involvement [172]. As dysarthria progresses, augmentative and alternative communication devices will be necessary, as oral motor exercises are not helpful [13,170,171,172,173].

#### 8.2.6. Weakness of the Respiratory Muscles

Failure of the muscles that support ventilation is the leading cause of death in ALS [174]. Diaphragmatic weakness first manifests as nocturnal hypoventilation and can lead to sleep disruption, increased anxiety, morning headaches, and excessive daytime fatigue. As the disease progresses, patients develop orthopnea, with inability to lie flat, dyspnea on exertion, and eventually, shortness of breath on sitting. A weak cough and difficulty in clearing secretions are associated symptoms. Patients may also develop a soft voice, as they need sufficient respiratory support to speak loudly [170,174].

### 8.3. Advanced ALS

The natural history of this pathology leads to generalized muscle weakness and eventually death, which mostly occurs due to ventilatory muscle failure. The continued involvement of a multidisciplinary team is essential to help the patient to cope with a worsening functional status [131,175,176,177].

## 9. Airway Clearance Strategies in ALS

### 9.1. Pathophysiology of Cough

The function of cough consists in clearing the airways of secretions and debris by generating a high-velocity airflow [178]. Cough can be of different types, such as voluntary, spontaneous, or induced. In addition, cough has three phases: inspiratory, compression, and expiratory. A large inspiration creates a large vocal cord opening followed by cessation of the airflow, glottis closure, and a high subglottic pressure effort. The duration of the compression phase varies and is affected by atypical abduction/adduction of the glottis and the respiratory muscle strength. The glottis again opens primarily under a high-velocity airflow with final sustained deceleration and ends with the expiratory airflow returning to baseline. Thus, air passes through the opening [178].

### 9.2. Cough Insufficiency/Cough Increase

Any alteration in this cough motor pattern will affect the efficacy of the effort. Silent aspiration is known as a lack of coughing in response to material entering the larynx [179]. Glottis adduction reduces the amount of subglottic pressure and thus the ability of the expiratory flow to clear the airways of unwanted substances. This coordinated airway closure generates pressure and prevents air escape during the compression phase of the cough maneuver [180]. The slow vocal cord abduction found in ALS patients results in significant atrophy in the posterior cricoarytenoid muscle, which affects cough expulsion [180,181].

Cough impairment in individuals with this pathology is attributed to the loss of upper and lower motor neurons according to an expert panel [179].

The goals of respiratory management care in ALS patients are to ensure a patent airway, prevent pulmonary infections, and control airway secretions. Any bulbar-type involvement decreases the peak cough flow and increases coughing focused on the movement of secretions through the proximal airways as the pathology progresses [181,182,183].

### 9.3. Manual Assistance

Assisted coughing and air packing are low-cost options that can be applied independently or with a trained caregiver. An assisted cough involves an abdominal thrust, which must be avoided after food intake, that allows for moving the diaphragm upward and increasing the expiratory flow [183]. As the peak cough flow decreases, assisted coughing becomes less effective in ALS patients. The aim of breathing techniques is to increase the inspiratory capacity and improve the peak cough flow [183].

#### Sputum Mobilization and Salivary Secretion Management

There are several multimodal strategies for airway clearance in patients with ALS, both proximal and peripheral. Identifying specific therapies early is critical to improving outcomes and quality of life for individuals with this pathology. The general goals of peripheral airway clearance techniques are increasing ventilation, improving mucus transport, loosening secretions [184].

As stated before, dysphagia and sialorrhea are common in ALS with bulbar involvement and will manifest as increased drooling and stagnation of oral secretions [185]. Because of these effects of these symptoms, including acute respiratory failure and death [186], treatments are based on cost-effective and sustained high-priority relief. Possessing an impaired swallow results in weight loss, dehydration, and malnutrition, thereby affecting the energy and strength needed to cough effectively [187]. Tongue movement, bulbar muscle atrophy, and laryngeal dysfunction are features of advanced ALS dysphagia [188].

## 10. ALS Forecast

The prognosis of ALS depends on the development of the pathology. Currently, the ALS Functional Rating Scale for ALS Revised (ALSFRS-R) is used for monitoring ALS progression [189,190]. One domain of this scale that gives prognostic information is respiratory function [99,191,192].

Although the median survival in ALS is only 2 to 4 years, there is a distribution of life duration that affects both the patient’s ability to understand the prognosis of the disease and the physician’s ability to discuss. However, the prognosis of the evolution of this disease is in its infancy, as the best models remain uncertain [193]. Therefore, clinical care teams must tell patients and their families about the progression of the pathology and the large number of symptoms that are expected, underscoring that predictions can vary [194,195].

## 11. Treatment

This disease is considered incurable. Treatment focuses on improving the quality of life of those affected using therapies that focus on modifying the disease. Considerable knowledge has been attained about the molecular pathology of ALS and the genetic background of the familial subtypes [196]. ALS intensely impairs day-to-day activities of daily living and health-related quality of life for both patients and caregivers [197,198].

### 11.1. Pharmacological Treatment

Riluzole, edaravone, and tofersen are drugs used in some countries to manage this pathology [199,200,201,202,203,204,205]; they are responsible for modifying the disease, but do not stop its progression. Therefore, muscle weakness remains unavoidable and leads to tetraplegia (paralysis of four limbs), dysarthria (difficulty in articulating words), swallowing dysfunction (feeling of food getting stuck in the throat), and chronic hypercapnic respiratory failure (clinical condition in which the patient has lower-than-expected arterial oxygen partial pressure and higher carbon dioxide partial pressure) [196,206,207].

Riluzole is an antiglutamate agent. It is responsible for improving the quality of life of patients in clinical trials and post-marketing analyses, although it remains controversial whether this event occurs in all stages of ALS or only in advanced stages of the disease [208,209,210]. This particular drug was the first to be approved by both the European Union and the U.S. Food and Drug Administration for the treatment of ALS. Its main mechanism of action is based on inhibiting toxic damage to neurons by the neurotransmitter glutamate through several pathways [211].

The antioxidant edavarone administered for 6 months was shown to be effective in a post hoc analysis of the first phase of three trials including patients who met the criteria of definite or probable ALS with duration of less than 24 months and ALSFRS-R subscale score >2 [212]. The trial was repeated, and it could be verified that edaravone slowed the pathology progression [212,213,214,215]. However, the use of this drug has not yet been approved worldwide for the reason that the safety and benefits of this drug are in a state of controversy [214].

In the United States, a mixture of dextromethorphan and quinidine was approved for use in cases of pseudobulbar involvement [216], and its main benefit is reflected in the reduction of crying and laughing episodes, as well as in an improvement in both social quality and quality of life [216]. However, this drug is not marketed in all countries, and there are alternative treatments that are more cost-effective, such as noninvasive ventilation, and improve the quality of life of ALS patients [216].

Recent advancements in ALS therapeutics have expanded the treatment landscape. AMX0035 (sodium phenylbutyrate/taurursodiol), a combination therapy targeting mitochondrial dysfunction and endoplasmic reticulum stress, demonstrated a 6.5-month survival benefit in the CENTAUR trial and received FDA approval in 2022 [215,217,218,219,220,221]. However, sodium phenylbutyrate/taurusodiol did not show benefits in the phase 3 PHOENIX trial and was ultimately discontinued by the manufacturer [222]. Additionally, stem cell-derived exosomes delivering neurotrophic factors (e.g., BDNF, GDNF) are under investigation for their neuroprotective effects in preclinical models [119,223].

### 11.2. Non-Pharmacological Treatment

In addition to drug therapy, there are other types of treatments for ALS such as stem cell therapy and gene therapy, among others. The recognition of the genetics and heterogeneity of ALS brings new therapeutic approaches to individuals suffering from ALS.

Gene therapy is a hopeful avenue for ALS treatment [207,224,225,226]. This therapy is based on four approaches to achieve the elimination of the toxic effects of known regulatory genes in this pathology [227]. They involve the use of complementary DNA or RNA sequences designed for the activation of RNA degradation, the use of small molecules to decrease the mutant protein load, the reversal of mutations to the wild-type forms in appropriate non-germ cells, and transcriptional interference [210,228,229].

In the brain and spinal cord of SOD1 rats, intracerebroventricular injections of SOD1-antibodies directly decreased SOD1 mRNA through RNase H activity, increasing the rats’ lifespan by 10 days. Intrathecally administered ASOs were well tolerated in humans, with no adverse effects [230,231,232].

Another important target is the *C9orf72* gene. Antisense oligonucleotides and other RNA interference-based approaches are being developed to reduce the expression of *C9orf72* mutant-related toxic products [233]. Several clinical trials are ongoing to assess the safety and efficacy of these therapies.

Gene replacement therapy is also being explored, particularly for genes like *FUS* and *TARDBP* (TDP-43), where loss-of-function mechanisms may contribute to disease pathogenesis. Gene-silencing therapies, such as ION363 for *FUS* mutations [234] and CRISPR-Cas9 approaches, are currently under research [30,234,235,236,237]. However, significant challenges remain in terms of delivery efficiency, specificity, and potential off-target effects [238].

Adeno-associated viral (AAV) vectors are commonly used to deliver functional copies of these genes to motor neurons. Preclinical studies have shown promising results in animal models [239,240].

Stem cell therapies are currently examined in phase 1, 2, or even 3 clinical trials using a variety of cell types. Novel stem cell approaches are primarily designed for neuroprotection. Mesenchymal stromal cells are used for their ability to secrete neurotrophic factors and modulate the immune system in ALS patients, two mechanisms that slow the pathology process in animal models [241].

Several studies have investigated the effect of therapeutic approaches using mesenchymal stem cells in mouse models of the disease, demonstrating that motor neuron loss was slower in the group treated with mesenchymal stem cells [242,243,244,245,246,247].

The therapeutic effect of mesenchymal stem cells has also been investigated in ALS patients through the intraspinal or intrathecal administration of bone marrow-derived mononuclear cells or fetal neural stem cells [248,249,250,251]. This demonstrates that much remains unknown about stem cell therapy, its mode of administration, and its clinical and safety endpoints [252,253,254,255,256].

However, some models of the disease have been challenged in recent years. This occurs because the results of animal models are not correctly translated into ALS patients. It is noteworthy that riluzole, the only approved neuroprotective treatment, showed a positive effect in ALS patients but no effect in a mouse model [257]. However, one of the predominant models for ALS is the SOD1 mouse model. Transgenic mice expressing a mutation in the SOD1 gene are undoubtedly very important for understanding the biological mechanisms of the disease. However, this model has failed due to methodological errors, as it has an inherent limitation. Therefore, the lack of a validated model for the disease is a real obstacle. Therefore, considerable efforts have been made to obtain cellular models of the disease from human induced pluripotent stem cells (hiPCS) [258]. These in vitro models guarantee a simple model of the disease and allow for the selection of candidate drugs, counting on approved drugs for their repositioning in ALS [259].

ALS treatments involve a comprehensive multidisciplinary approach to improve the quality of life and survival of these patients. This approach includes a group of health professionals such as neurologists, physiotherapists, nurses, and social workers, who utilize a problem-solving-based education process, specifically prioritizing physiotherapy, occupational therapy, and speech therapy that focus on maximally engaging the activity and participation of ALS patients [210].

#### 11.2.1. Physiotherapy as Multidisciplinary Care

Currently, the American Academy of Neurology advises the early referral of individuals with ALS to a multidisciplinary clinic [260], as this can assess and coordinate their rehabilitation needs [261]. It is considered that the rehabilitation of these patients will allow for progress in their independence in a safe way, controlling their symptoms, and thus achieving a better quality of life. Therefore, physiotherapy is essential throughout the course of the disease. In the early stages, the goal will be to try to slow down the loss of mobility and respiratory function, and in the advanced stages, it will be to try to alleviate the effects of immobility. The benefits of physiotherapy for this type of patients range from avoiding retractions and pain related to mobility to preventing and acting on respiratory problems and also teaching the family and caregivers the correct management of the patient [155,176,262,263].

The exercises performed with this type of patients are determined by the disease stage in which they are. In the initial stages, aerobic exercises or exercises in which the patient himself performs the movement are usually performed. As the disease progresses, the exercises become more passive, and therefore mobilizations or stretching are performed [147,176,183,198].

Both inspiratory muscle training, manual cough assistance, and lung volume recruitment by air stacking are considered specific respiratory care interventions and address the relief of respiratory failure symptoms [264].

#### 11.2.2. Exercise

Exercise is effective in delaying respiratory failure in patients with this pathology [265,266], as it preserves the respiratory and limb function. These patients benefit from increased breathing during exercise because there is increased activation of the respiratory motor neurons or muscles [266].

Muscle weakness is one of the most common symptoms in a person with ALS [7,267,268]. A weak muscle can be damaged if it is overworked, because it can reach its maximum limit. Consequently, some experts believe that ALS patients should not engage in an exercise program. However, if a person with this pathology is not active, there is a loss of muscle performance and weakness due to lack of muscle use, in addition to the deconditioning and weakness inherent to the pathology. If the reduced level of activity is persistent, it can lead to the deterioration of organ systems. In addition, patients with ALS develop muscle weakness, which results in muscle and joint stiffness, leading to contractures and pain. All of this makes the activities of daily living more difficult to perform [7,267,268].

Therefore, range-of-motion, aerobic, resistance, and combined interventions can be performed with these ALS patients, in addition to breathing and balance exercises. Regarding the range-of-motion exercises, it is essential to be able to maintain a certain range of motion in all stages of ALS, improving mobility, self-care, posture, and wheelchair seating and reducing pain and the risk of injury. Range-of-motion exercises are essential in this pathology due to the loss of strength and function in these patients [269].

Guiding patients and teaching them the care they should possess through a passive or active telehealth range-of-motion program is feasible and affordable. Both caregivers and patients benefit from detailed instructions on stretching techniques and dosage, as well as hand positioning [270].

Regarding aerobic exercises, it must be considered that patients with ALS have reduced aerobic capacity related to the loss of lean muscle mass, which can deteriorate due to physical deconditioning [271]. Fatigue, decreased activity tolerance, and impaired function are the secondary symptoms most frequently present in this pathology population, resulting in reduced efficiency and aerobic capacity.

Regarding resistance exercises, it must be considered that this type of exercises are prescribed because of injury avoidance, avoidance of muscle strengthening exercises involving less force than antigravity exercises [131,151,269,272], and avoidance of eccentric strengthening exercises [273,274].

Really, combined interventions produce better results than individual exercises, as they have positive effects on pain reduction, function, and quality of life [275,276]. Furthermore, breathing exercises performed by physiotherapists on ALS patients, focused on lung volume recruitment, muscle strengthening based on inspiratory and expiratory muscle training, and airway clearance techniques, improve hospitalization, survival, or the time before the initiation of mechanical ventilation [277].

## 12. Palliative Care

Euthanasia is one of the most controversial topics in contemporary bioethics. Very few countries have adopted the radical approach of accepting the termination of life by a physician. Most countries adhere to its prohibition. This increases the likelihood that the controversy will center on withholding or withdrawing medical treatment, especially life-sustaining treatment [278].

Among the different patterns of communication that exist between an ALS patient and a healthcare professional, communication may be avoided or delayed, an individual may consider that the best solution is to die and actively seek help, or a healthcare professional may ignore or disregard the patient’s wishes or respect them.

Since communication about disease progression is mostly accompanied by feelings of loss, fear, and grief [175,279,280], communication with the patient is sometimes delayed or avoided. Most of the time, a wait-and-see strategy is followed [279,281]. A large number of patients cling to positive thoughts, try to live day by day, and do not want to think about the consequences that the disease may bring.

Some patients show their wish to die to both family and healthcare professionals. In some cases, the patient’s wishes to die or to withdraw from treatment are ignored. In other cases, the family members do not accept the individual’s will and do not support him or her [282,283].

Existing research suggests that options such as prolonging or shortening an individual’s life by avoiding or delaying communication or decision-making or simply ignoring the patient’s will are not compatible with the ethical principles associated with autonomy and non-maleficence [148]. Thus, these strategies cannot be ethically justified even though communication is an obstacle, and a time-consuming investment is required. Health professionals should apply the existing guidelines to address patients’ dying wishes. In countries where these guidelines have not yet been developed, it is necessary to advance this debate with discourses at the national, regional, and international level to obtain good communication methods and to expand good practices, so that the patient’s opinion is respected and the patient is aware of the evolution of the disease, of the adverse effects that will occur as this pathology is prolonged over time, and finally of its evolution until death. Therefore, once the patient has been informed of the complexity of this disease, he/she can make a free choice about his/her life [284].

## 13. Conclusions

This review on ALS underscores the inherent complexity of this neurodegenerative disease, from its phenotypic heterogeneity to the intricate network of pathophysiological mechanisms that contribute to its progression. ALS manifests as a diagnostic challenge due to its clinical variability, requiring a comprehensive approach that combines a detailed neurological evaluation with complementary tests to confirm upper and lower motor neuron involvement. The application of standardized diagnostic criteria, such as the revised El Escorial criteria, facilitates a more accurate classification of the disease, which in turn allows for better patient stratification and more informed therapeutic decision-making.

Pathophysiology research has revealed the involvement of multiple molecular and cellular pathways in ALS, including alterations in autophagy, RNA metabolism, nucleocytoplasmic transport, and protein aggregate formation. Genes such as *C9orf72*, *SOD1*, TDP-43, and *FUS* play a crucial role in these pathological processes, and their dysfunction contributes to motor neuron degeneration and disease progression. Understanding these underlying mechanisms is critical for the development of targeted therapies that can modify the course of ALS and improve the clinical outcomes.

Treatment of ALS remains a challenge, but advances in multidisciplinary care and the development of new drugs offer hope for patients and their families. Riluzole, edavarone, and tofersen are approved treatments that have been shown to modestly prolong survival in some ALS patients. However, their efficacy is limited, and more effective therapies are urgently needed. A multidisciplinary approach, including physiotherapy, occupational therapy, speech therapy, and psychosocial support, is essential to optimize patients’ quality of life and address the multiple symptoms and complications of the disease.

The management of specific symptoms, such as dysphagia, dysarthria, cramping, and sleep disturbances, requires an individualized approach and the application of specific strategies, such as airway clearance techniques, noninvasive ventilation, and neuropathic pain management. The prognosis for ALS remains variable, but ongoing research and advances in clinical care offer promise for improving the quality of life and prolonging the survival of patients affected by this devastating disease. It is critical to continue to promote translational research and the development of innovative therapies that address the underlying causes of ALS and offer new treatment options for this complex and challenging disease.

## Abbreviations

The following abbreviations are used in this manuscript:

ALS	Amyotrophic Lateral Sclerosis
FTD	Frontotemporal Dementia
C9orf72	Chromosome 9 Open Reading Frame 72
TDP-43	TAR DNA-Binding Protein 43
SOD1	Superoxide Dismutase 1
FUS	Fused in Sarcoma
NfL	Neurofilament Light Chain
pNfH	Phosphorylated Neurofilament Heavy Chain
GFAP	Glial Fibrillary Acidic Protein
CSF	Cerebrospinal Fluid
MRI	Magnetic Resonance Imaging
NIV	Non-Invasive Ventilation
FDA	Food and Drug Administration
RNA	Ribonucleic Acid
DNA	Deoxyribonucleic Acid
miRNA	MicroRNA
BDNF	Brain-Derived Neurotrophic Factor
GDNF	Glial Cell Line-Derived Neurotrophic Factor
AMX0035	Sodium Phenylbutyrate/Taurursodiol
ALSFRS-R	ALS Functional Rating Scale-Revised
SIMOA	Single-Molecule Array
